# Effect and Mechanism of *EGFL7* Downregulation in Human Osteosarcoma Cells on the Biological Function of Co-cultured HUVEC

**DOI:** 10.4274/balkanmedj.2017.0045

**Published:** 2018-03-15

**Authors:** Xia Li, Li-Feng Liu, Yang-Zhou Liu, Yu-Tao Pan, Guang Li, Qing-You Lu, Zeng-Chun Li

**Affiliations:** 1 Graduate School of the Second Military Medical University, Shanghai, China; 2 Department of Emergency Trauma, Shanghai East Hospital, Shanghai, China

**Keywords:** Adhesion, angiogenesis, co-culture, epidermal growth factor-like domain 7, migration, osteosarcoma

## Abstract

**Background:**

Even though epidermal growth factor-like domain 7 is known to be overexpressed in osteosarcoma and is associated with poor clinical outcome, few reports are available regarding its mechanism.

**Aims:**

The objective of this study was to explore the effect and mechanism of downregulating epidermal growth factor-like domain 7 expression in a human osteosarcoma cell line on the biological function of co-cultured human umbilical vein endothelial cells.

**Study Design:**

Cell study.

**Methods:**

In the present study, human osteosarcoma cell lines U2OS, Saos-2, HOS, and MG63, and normal human osteoblasts were cultured in Dulbecco’s Modified Eagle Medium containing 10% fetal bovine serum and 1x antibiotics at 37 °C and 5% CO_2_ in an incubator. Of the four osteosarcoma cell lines, U2OS expresses the highest level of epidermal growth factor-like domain 7 mRNA as determined using quantitative reverse transcription polymerase chain reaction. With the knockdown of epidermal growth factor-like domain 7 in U2OS and human umbilical vein endothelial cells by lentivirus, the proliferation and apoptosis of U2OS and human umbilical vein endothelial cells were investigated using MTT and flow cytometry assays. After the co-culture of human umbilical vein endothelial cells and epidermal growth factor-like domain 7-knockdown U2OS, the *in vitro* effects on cell proliferation, apoptosis, adhesion, migration, and the angiogenic ability of human umbilical vein endothelial cells were detected using MTT, flow cytometry, Transwell, and tube formation assays, respectively. The expressions of phosphoinositide 3-kinase, phospho-Akt, total Akt, and vascular endothelial growth factor in human umbilical vein endothelial cells were detected using western blot assay.

**Results:**

Lentivirus with epidermal growth factor-like domain 7 shRNA could not significantly affect the proliferation and apoptosis of both U2OS and human umbilical vein endothelial cells, whereas the knockdown of epidermal growth factor-like domain 7 in U2OS could significantly inhibit the migration, adhesion, and angiogenic ability of co-cultured human umbilical vein endothelial cells. In addition, the expressions of phosphoinositide 3-kinase, phospho-Akt, and vascular endothelial growth factor in human umbilical vein endothelial cells decreased after co-culturing with epidermal growth factor-like domain 7-knockdown U2OS.

**Conclusion:**

Epidermal growth factor-like domain 7-knockdown U2OS cells inhibit the migration, adhesion, and angiogenesis of co-cultured human umbilical vein endothelial cells by diminishing phosphoinositide 3-kinase, Akt signaling pathway activity and vascular endothelial growth factor expression.

Osteosarcoma is the most frequently occurring solid bone cancer with the second highest cancer-related mortality in pediatric patients (1). Even though advancements in cancer treatment have improved the cure rate for osteosarcoma, its 5-year survival rate has not improved since the last 40 years, and traditional chemotherapy and surgical treatments have reached their therapeutic limits. In fact, for patients who are at the highest risk for tumor recurrence and metastasis, which have been the leading causes of mortality, the long-term survival rate is lower than 20% with poor prognosis. Hence, exploring novel options for clinical therapy becomes imperative. While novel anticancer therapy targets are essential strategies for osteosarcoma treatment, anti-angiogenesis has gained prominence as a novel approach for cancer treatment besides surgery, radiotherapy, and chemotherapy (2). The growth of blood vessels within a tumor is a critical factor that accounts for the rapid increase in solid tumor size, local invasion, and metastasis, and is also a prognostic indicator of the development of osteosarcoma. Reports have established the efficacy of anti-angiogenesis inhibitors, such as sorafenib and bevacizumab, in inhibiting tumor growth in osteosarcoma by conducting research on animals (3) and clinical therapy (4,5).

Epidermal growth factor-like domain 7 (EGFL7) is secreted by the endothelium of blood vessels, and its expression increases with the development of tumor growth and metastasis. In both physiological and pathological processes, EGFL7 plays a vital role in the formation of lumens and in the functions of new blood vessels. The inhibition of EGFL7 expression can prevent lumen formation and induce the loss of function of blood vessels, thereby suppressing tumor growth and metastasis. Recently, EGFL7 has become a novel potential target against cancer. Luo et al. (6) demonstrated that the elevated EGFL7 expression correlated with poor clinical outcomes, such as advanced tumor stage and recurrent and metastatic osteosarcoma (2). Even though research supports EGFL7 as a potential prognostic marker for the diagnostics and therapeutics of osteosarcoma, few studies have explored the underlying mechanism of EGFL7 in osteosarcoma. The epidermal growth factor receptor (EGFR) binding with two internal EGF-like domains within an EGFL7 protein can activate the EGFR signaling pathway. Several reports have demonstrated that EGFR is expressed at high levels in human osteosarcoma (7). In addition, certain clinical reports have established that EGFR antibody could aid in the treatment of advanced osteosarcoma (8). Hence, we hypothesize that EGFL7 affects the EGFR downstream signaling pathway. Reportedly, mitogen-activated protein kinase (MAPK), phosphoinositide 3-kinase (PI3K), and jun N-terminal kinase (JNK) are downstream molecules of the EGFR signaling pathway, and reports have indicated a positive correlation between EGFR and Akt expression (9). Furthermore, overexpressed EGFL7 activates the RAS-MAPK and PI3K-Akt signaling pathways (10,11), which, in turn, regulate the pathological development of osteosarcoma according to clinical (12) and laboratory reports. In Jeg3 of human choriocarcinoma cell line, EGFL7 overexpression could increase approximately two-fold higher levels of the phosphorylated forms of Akt (10). Moreover, a change in phospho-Akt (13) leads to tumor metastasis, invasion, and adhesion, as well as the activation of vascular endothelial growth (VEGF), for promoting angiogenesis in osteosarcoma (14,15). In addition, VEGF has been reported to play an essential role in tumor angiogenesis and is considered as an effective biomarker of prognosis in patients with osteosarcoma. Hence, whether EGFL7 affects the growth and metastasis of osteosarcoma through the PI3K-Akt signaling pathway and VEGF remains unclear. With the correlation of high EGFL7 expression with osteosarcoma malignancy, all data indicate that the expression of these pathway molecules may be associated with EGFL7, and that tumor growth and metastasis could be affected by the combination of EGFL7 and pathway molecules by regulating the tumor microvascular density (MVD). Using an *in vitro* co-culture system, this study aimed to simulate the interaction between tumor cells and endothelial cells *in vivo* and to investigate the effect and the underlying mechanisms of EGFL7 expression in both cell types.

## MATERIALS AND METHODS

### Cell culture

Human osteosarcoma cell lines U2OS, Saos-2, HOS, and MG63, and normal human osteoblasts (NHOst) were obtained from the Cell Center of the Chinese Academy of Sciences and cultured in Dulbecco's Modified Eagle Medium (Invitrogen, Carlsbad, CA) containing 10% FBS (Invitrogen) and 1x antibiotics at 37 °C and 5% CO_2_ in an incubator.

Reverse transcription quantitative polymerase chain reaction experiments

After culturing, NHOst, U2OS, Saos-2, HOS, and MG63 cells were subjected to total RNA extraction using TRIzol reagent (CW Biotech, Beijing, China). Then, the RNAs were subjected to quantitative detection with NanoDrop. We used 1 μg of the total RNA from each sample for reverse transcription, using random primers, followed by reverse transcription-quantitative polymerase chain reaction (RT-qPCR) amplification. In the present study, primer sequences for EGFL7 and glyceraldehyde-3-phosphate dehydrogenase (GAPDH) were as follows: EGFL7 forward: 5’-GATGGCGGGGTGACACT-3’, EGFL7 reverse: 5’-CACTGTCCACTCCTGTCGGG-3’; GAPDH forward: 5’-CGGAGTCAACGGATTTGGTCGTAT-3’, GAPDH reverse: 5’-AGCCTTCTCCATGGTGGTGAAGAC-3’. The conditions for amplification were as follows: pre-denaturation at 94 °C for 3 min; 40 cycles of denaturation at 94 °C for 30 s, annealing at 57 °C for 30 s, and extension at 72 °C for 2 min, followed by one cycle of extension at 72 °C for 10 min.

### Building lentivirus containing EGFL7 shRNA and cell infections

On the basis of the GenBank database of *EGFL7* gene nucleotide sequences, EGFL7 shRNA sequences were selected for designing and restructuring of the lentivirus vector containing green fluorescent protein. After packing in 293T cells, virus particles were formed containing EGFL7 shRNA (named Lenti-EGFL7/shRNA), followed by the determination of its titer. After achieving a cell confluence of 30%, cultured cells in the logarithmic phase were infected with Lenti-EGFL7/shRNA or blank virus particles. Meanwhile, we added 8 μg/mL of polybrene for enhancing infection. After 48 h, cells were observed under fluorescence, and EGFL7 mRNA expression was investigated.

### Cell proliferation assay

According to the groups mentioned above, after 48 h, the infected cells were prepared to be single-cell suspensions, inoculated in 96-well plates with a density of 2.000/well, and cultured for 24-72 h. In the 24-well co-culture system (0.4-μm aperture), HUVEC were seeded into the lower chamber with 8,000/well and U2OS/EGFL7i into the upper chamber with 100,000/well. After 24-72 h, 10 μL of MTT 0.5 mg/mL was added to each well, and then values were recorded at 492 nm absorbance.

### Cell apoptosis assay

After U2OS or HUVEC were infected in the 6-well co-culture system (0.4-μm aperture) for 48 h, HUVEC were seeded into the lower chamber with 100,000/well and U2OS/EGFL7i into the upper chamber with the same density, followed by co-culturing for 48 h. Before testing, cells were trypsinized, centrifuged, and counted, adjusting to 1,000,000 cells/mL. In accordance with the manufacturer’s instructions (Dojindo, Kumamoto, Japan), 100 μL of the cell suspension was obtained from each well, to which we added Annexin V-fluorescein isothiocyanate conjugate, kept away from light for 30 min at room temperature, added PI, kept away from light for 15 min at room temperature, and, finally, added 400 μL of Annexin V binding solution.

### Cell migration assay

In the 24-well Transwell (8.0-μm aperture) co-culture system, HUVEC were seeded into the upper chamber with 100,000/well and U2OS/EGFL7i or U2OS into the lower chamber with the same density. After 24 h, the chamber membrane was washed with PBS and stained with crystal violet for 1 h. Then, cells on the upper side of the filter membrane were carefully wiped with cotton swabs. After washing with PBS, migrated cells on the bottom side of the membrane were observed using an optical microscope.

### Transwell assay for cell adhesion test

While U2OS/EGFL7i or U2OS were seeded into the upper chamber, HUVEC were seeded into the lower chamber with co-culture for 48 h. After trypsinization and preparation of single cell suspension of co-cultured HUVEC, 10,000/well HUVEC were seeded into a new 96-well plate coated with rat tail collagen. The supernatant and suspended cells were discarded after 60 min, and the absorbance of adherent cells in the well was detected using MTT assay.

### Tube formation of human umbilical vein endothelial cells *in vitro*

In the 24-well Transwell (0.4-μm aperture) co-culture system, 300 μL of Matrigel was added into the upper chamber with incubation for 1 h at 37 °C. Then, HUVEC were seeded into the upper chamber with 50,000/well and U2OS/EGFL7i or U2OS cells were seeded into the lower chamber with 100,000/well for co-culture for 48 h. The tube formations of HUVEC were observed using an optical microscope.

### Western blot assay

In the 24-well Transwell (0.4-μm aperture) co-culture system, U2OS/EGFL7i or U2OS were seeded into the upper chamber, and HUVEC were seeded into the lower chamber. After 48 h, HUVEC were lysed in the cell lysis buffer. The total protein content was measured using Coomassie brilliant blue assay. In addition, an equal amount of protein was electrophoresed on 10% sodium dodecyl sulphate-polyacrylamide gel electrophoresis and transferred to PVDF membranes. The membranes were blocked, incubated for 2 h with primary antibodies, and subsequently incubated with secondary antibody (Boster, Wuhan, China) for 2 h. The blots were then visualized using ECL detection reagents (Beyotime, Shanghai, China). We used the following antibodies: PI3K, p-Akt, Akt, VEGF, and GAPDH (Cell Signaling Technology, MA).

### Statistical analysis

All statistical analyses were performed using the SPSS19.0 software package (SPSS Inc., Chicago, IL). Results are shown as mean ± standard deviation. In addition, Student’s t-test was used for determining the statistical difference between experimental and control groups. We considered p<0.05 as statistically significant.

## RESULTS

### Relatively high expression of EGFL7 in U2OS

**Figure 1:**
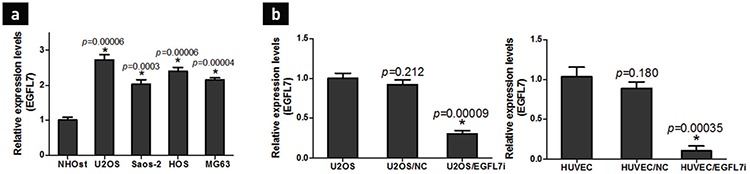
Analysis of EGFL7 mRNA expression and verification of the knockdown efficiency by Lenti-EGFL7/shRNA. Reverse transcription-quantitative polymerase chain reaction analysis of EGFL7 mRNA expression in human osteosarcoma cell lines U2OS, Saos-2, HOS, and MG63, and normal human osteoblasts, NHOst. The relative gene fold changes were normalized by GAPDH and then by EGFL7 level of NHOst. Data are presented as mean ± standart deviation (n=3). *p<0.05 compared with NHOst (a), EGFL7 gene silencing by Lenti-EGFL7/shRNA into U2OS and HUVEC for 48 h. The RNA was extracted and reverse transcription-quantitative polymerase chain reaction was performed. The knockdown effects were determined with normalization to native cell control. *p<0.05 compared with cell control (b).

For the subsequent EGFL7 knockdown experiment, NHOst, U2OS, Saos-2, HOS, and MG63 were selected for identifying the cell line with the higher expression of EGFL7 using RT-qPCR and comparing the relative EGFL7 expression with NHOst. Four osteosarcoma cell lines present different cell morphologies with epithelial morphology for U2OS, Saos-2, HOS, and fibroblast morphology for MG63. The results revealed that EGFL7 was expressed in all five cell lines, and the comparison with NHOst revealed that EGFL7 expression increased in all four osteosarcoma cell lines. These findings acted as reminders regarding the potential high expression of EGFL7 in osteosarcoma but not in normal tissues (Figure 1a). Furthermore, despite a similar gene level of EGFL7 in all four osteosarcoma cell lines, U2OS exhibited a relatively higher trend for the EGFL7 expression. Hence, U2OS was selected as the experimental cell line.

Inhibition of lenti-EGFL7/shRNA for EGFL7 mRNA expression Figure 1b shows that the EGFL7 mRNA expression was detected using RT-qPCR in both U2OS and HUVEC; however, the EGFL7 mRNA expression of the silenced group was significantly reduced in comparison with the no transfection and negative transfection groups (p<0.05). In addition, the difference between the negative transfection group and the no transfection group was not noticeable (p>0.05), which suggests that Lenti-EGFL7/shRNA successfully inhibited the EGFL7 mRNA expression of U2OS and HUVEC.

### No evident effect of *EGFL7* gene silencing on U2OS proliferation and apoptosis

**Figure 2:**
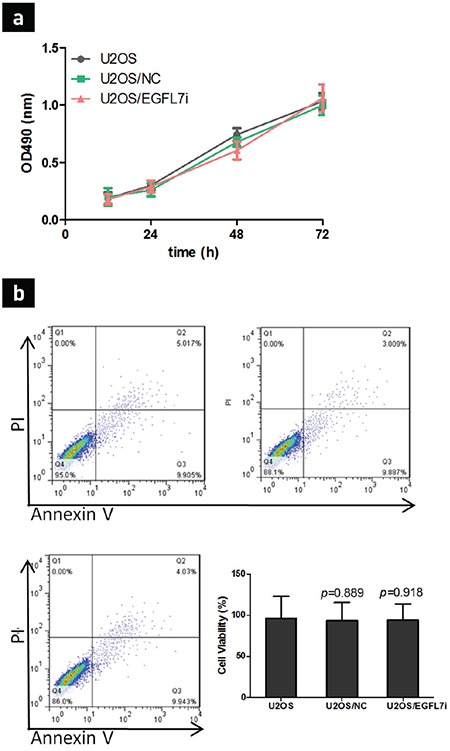
The effect of EGFL7 on U2OS proliferation and apoptosis. U2OS was incubated with different shRNA, as described in Materials and Methods, and then cells proliferation was detected using MTT assay after 24, 48, and 72 h growth (a), U2OS apoptosis after EGFL7 knockdown for 48 h was assessed using FACS assay. The percentage of viable U2OS was shown as mean of three independent experiments ± standard deviation (b).

The comparison of the U2OS negative transfection group with the no transfection group did not reveal any evident statistical difference (p>0.05; Figure 2a). These results indicated that the lentivirus system exerted no significant effect on U2OS proliferation, and compared with the no transfection group, variations in the U2OS/EGFL7i group’s proliferation were also not significantly different. These results illustrated that *EGFL7* gene silencing did not succeed in inhibiting bone sarcoma U2OS proliferation. When apoptosis was detected using the flow cytometry assay, *EGFL7* gene silencing exerted no effect on the apoptosis of U2OS compared with the no transfection and negative transfection groups (Figure 2b). These results suggest that the mechanism of EGFL7 promoting osteosarcoma does not play a role in the proliferation and apoptosis of tumor cells.

### No evident effect of *EGFL7* gene silencing on HUVEC growth and apoptosis

**Figure 3:**
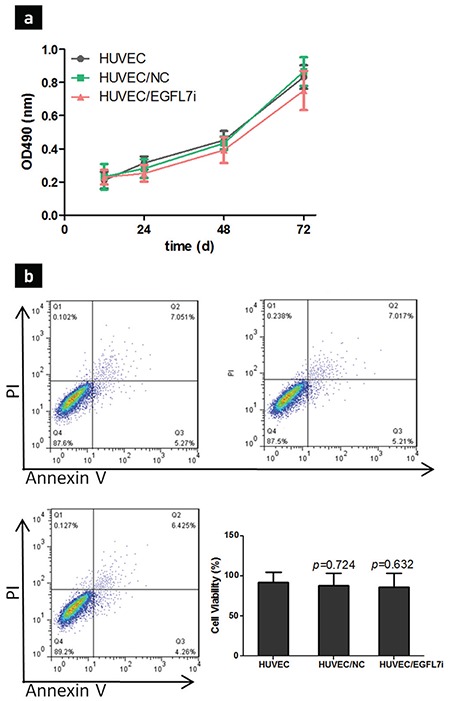
The effect of *EGFL7* on HUVEC cell proliferation and apoptosis. HUVEC were incubated with different shRNA, as described in Materials and Methods, and then cells proliferation was detected using MTT assay after 24, 48, and 72 h growth (a), HUVEC apoptosis after *EGFL7* knockdown for 48 h was assessed using FACS assay. The percentage of viable HUVEC was shown as mean of three independent experiments ± standard deviation (b).

We observed no evident effect on the HUVEC growth and apoptosis with *EGFL7* gene silencing (Figure 3a, 3b), which suggests that *EGFL7* genes may not affect endothelial cell proliferation and apoptosis in tumors, possibly through another pathway.

### EGFL7 inhibition of U2OS affects the HUVEC in co-culture system

**Figure 4:**
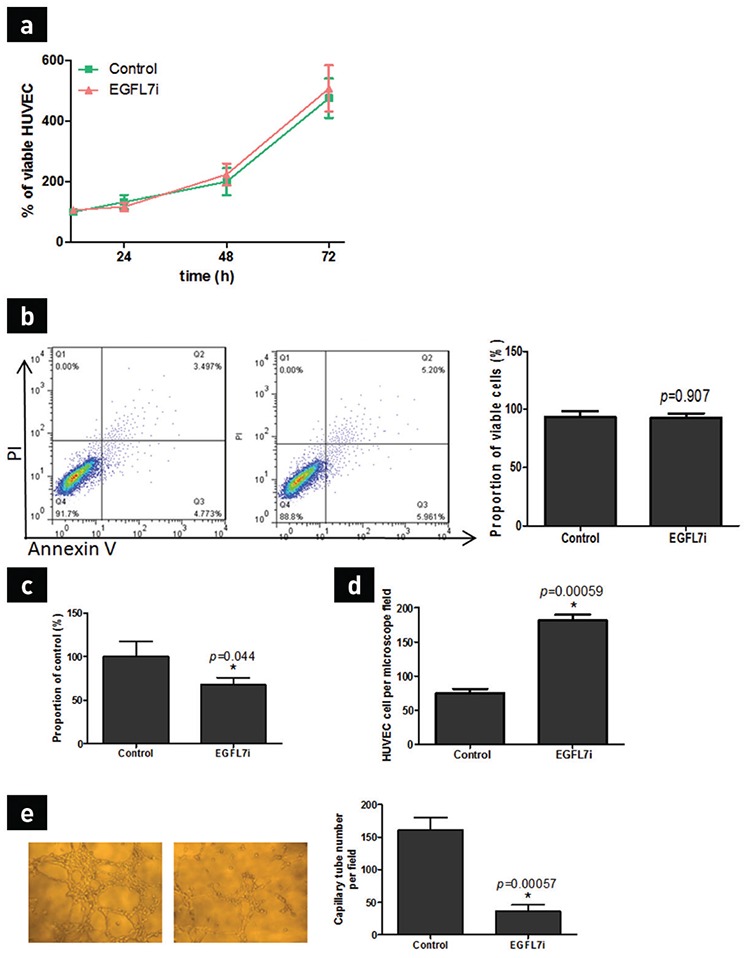
The effect of *EGFL7* gene downregulation of U2OS on HUVEC. HUVEC proliferation following co-culture with U2OS/EGFL7i or U2OS, as assessed using MTT assay after 24, 48, and 72 h co-culture (a), HUVEC were co-cultured with U2OS/EGFL7i or U2OS for 48 h and the apoptosis was determined using FACS assay (b), the adhesion of HUVEC was assessed by putting HUVEC in 96-well plate coated with rat tail collagen for 60 min after co-culturing with U2OS/EGFL7i or U2OS cells for 48 h. Then, the ODs were detected with MTT (c), the migratory ability of HUVEC was assessed using the Transwell migration assay after 48 h co-culturing with U2OS/EGFL7i or U2OS (d), HUVEC formed vessel-like structures (tubes) following incubation with U2OS/EGFL7i or U2OS. The vessel tube number was calculated from several microscope fields. All experiments were performed in triplicates. Data are shown as mean ± standard deviation. *p<0.05 compared with U2OS control (e).

Even though *EGFL7* gene inhibition of U2OS reduced HUVEC’s adhesion, migration, and microtubule formation, it did not affect the proliferation and apoptosis. We observed no significant difference in HUVEC’s proliferation and apoptosis in the two groups of U2OS/EGFL7i or U2OS co-cultured cells (p>0.05; Figure 4a, 4b). However, compared with the U2OS co-cultured cells, HUVEC microtubule formation, cell migration, and adhesion were noted to be reduced in the U2OS/EGFL7i cell co-culture group.

### Involvement of the PI3K-Akt pathway in HUVEC functional changes

**Figure 5:**
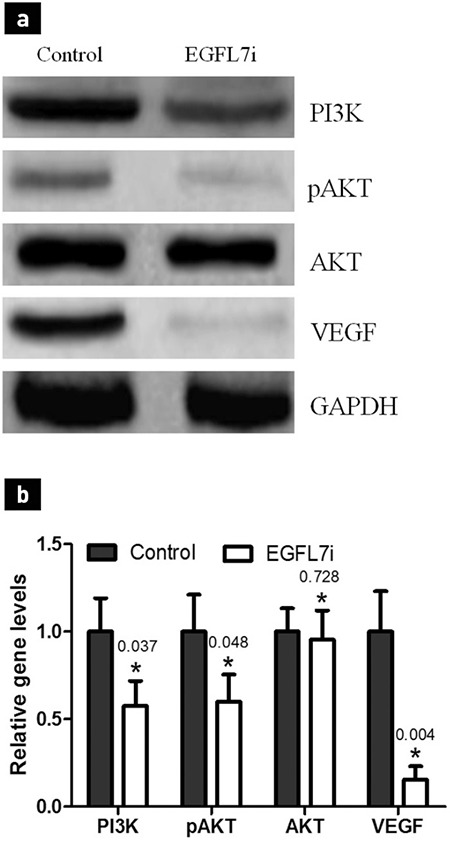
The PI3K-Akt expression of HUVEC in the co-culture system, co-cultured with U2OS/EGFL7i for 48 h, the expression of PI3K, p-Akt, and VEGF in HUVEC declined compared with U2OS co-cultured group. Representative images have been exhibited (a), grayscale scan for protein expression. *p<0.05 compared with U2OS control (b).

Findings of the western blot assay revealed a significant down-expression of PI3K in HUVEC after HUVEC were co-cultured with U2OS or U2OS/EGFL7i cells for 48 h, whereas we noted a decrease in its downstream effector, p-Akt (Figure 5a). However, no variations were observed in the total Akt protein level. These results revealed that the EGFL7 suppression in osteosarcoma cells affected the PI3K-Akt pathway in HUVEC. In addition, the VEGF of HUVEC decreased, which suggests that the decline in VEGF may be associated with the decrease in HUVEC migration, angiogenesis, and adhesion.

## DISCUSSION

This study explored the following: (1) downregulation of EGFL7 did not affect the proliferation and apoptosis of human osteosarcoma cell line U2OS; (2) it also did not affect the proliferation and apoptosis of HUVEC; (3) the *in vitro* co-culture transwell system was used for simulating the interaction between two cells *in vivo*, and the inhibited EGFL7 in U2OS was able to significantly reduce the migration, adhesion, and angiogenesis of HUVEC; (4) combined with these functional changes, the expression levels of PI3K, p-Akt, and VEGF decreased. Recent reports have demonstrated that while EGFL7 affected cell migration, it shows unremarkable effects on proliferation. For example, the proliferation of smooth muscle cells is not affected by EGFL7 without affecting the formation of the existing blood vessels, whereas EGFL7 prevents the apoptosis of smooth muscle cells for maintaining the stability of the blood vessels. However, literature is not consistent regarding the effect of EGFL7 on cell proliferation. Several reports have determined that the proliferation and cell-cycle distribution of human pancreatic cancer cells (16), human choriocarcinoma cells (10), and renal cell carcinoma (17) are not affected by EGFL7. Moreover, EGFL7 only presents the short-term inhibition of HUVEC’s proliferation without affecting the long-term growth (18,19). In this study, the proliferation and apoptosis of both cell types exhibited no variation after EGFL7 shRNA interference with U2OS and HUVEC, which indicates that EGFL7 may not affect cell proliferation, and there may be other EGFL7 mechanisms affecting tumor growth. Furthermore, using the Transwell system, the adhesion, migration, and angiogenesis of HUVEC were down regulated after EGFL7 knockdown in U2OS, which suggests that EGFL7 may influence tumor growth and metastasis by affecting the angiogenesis of endothelial cells. As an endothelial cell-specific secreted protein, EGFL7 expression is high during embryonic development, whereas a low level of expression is maintained in the adult body, except for a higher level of expression in vessel-rich organs, indicating the crucial role played by EGFL7 in angiogenesis. Furthermore, several reports have demonstrated uncontrolled EGFL7 expression in tumors (20). Parker et al. (21) reported that EGFL7 correlated with lumen formation, which is a key step in neovascularization under both physiological and pathological conditions, and played an essential role in the function. In addition, Luo et al. (6) established that the EGFL7 level positively correlated with osteosarcoma classification, prognosis, and metastasis, highlighting its potential as a biomarker for tumor prognosis. Furthermore, with the exception of the vascular endothelial cells in solid osteosarcoma tumor, EGFL7 is significantly expressed in tumor cells. Put together, these findings reflect that EGFL7 may be critical for tumor growth and metastasis. However, to our knowledge, few reports have explored the underlying mechanism of EGFL7 in osteosarcoma. The findings of this study demonstrated that EGFL7 knockdown causes the downregulation of the PI3K-Akt pathway. EGFL7 activates EGFR-related pathways by two EGF-like domains (22,23); this is consistent with the results of certain clinical studies that demonstrated that EGFR has been clinically detected to have high levels of expression in human osteosarcoma cells (7) and can be verified as a therapeutic target in osteosarcoma (24). Moreover, EGFL7 is upregulated in osteosarcoma. Reportedly, the PI3K-Akt is the most essential cancer-causing pathway in humans (25), which is excessively activated in osteosarcoma (25) and causes a series of downstream changes, including invasion, angiogenesis, metastasis, and so on. Even though few studies explain the relationship between osteosarcoma, EGFL7, and PI3K-Akt, the association of EGFL7 with PI3K-Akt has been established in other types of tumors. Shena et al. (10) determined that overexpressed EGFL7 in human trophoblast cells can activate the PI3K-Akt pathway. Similarly, Luo et al. (11) determined that the expression of p-Akt can be enhanced by EGFL7 overexpression in gastric cancer cells. Both results are consistent with the findings of this study, which suggests that the activation of the PI3K-Akt pathway in tumor cells may be the common mechanism in EGFL7 promoting the growth and metastasis of the tumor. However, further extensive research is warranted for obtaining a deterministic correlation conclusion.

VEGF is one of the crucial angiogenesis regulatory factors, which can effectively promote the proliferation of vascular endothelial cells. In the endothelium, VEGF specifically affects the VEGF receptor, including stimulating the proliferation of endothelial cells and increasing the permeability of blood vessels for promoting tumor growth. Zhao and Qiu (26) determined that the VEGF expression correlates positively with the MVD in osteosarcoma. The complicated regulatory mechanisms of VEGF include various signal molecules regulating the transcription and expression. Moreover, the secretion of VEGF can affect the angiogenesis and endothelial cell migration by binding to the VEGF receptor on the membrane surface of endothelial ells. Reports on other types of tumors revealed that the PI3K-Akt pathway is one of the mechanisms regulating VEGF, such as via mammalian target of rapamycin, hypoxia-inducible factor-1α (27) for regulating angiogenesis (28). In this study, we established that co-cultured U2OS/EGFL7i cells led to the decrease in VEGF expression in HUVEC, which indicated the involvement of PI3K-Akt pathway in the process. Meanwhile, the decrease in VEGF can mediate the adhesion, migration, and angiogenesis ability of HUVEC. Overall, to the best of our knowledge, this is the first study that investigates the mechanism of EGFL7 in a co-culture system mimicking osteosarcoma, and we determined that EGFL7 siRNA interfered with osteosarcoma tumor cells and could attenuate the migration, adhesion, and angiogenesis of HUVEC with the participation of the PI3K-Akt pathway and VEGF. Hence, owing to the clinical correlation of EGFL7 with osteosarcoma and its target potential for therapy, its mechanism is worth further research.
